# Genome-Wide Identification of *RTE* Gene Family Members in Sweet Potato and Their Expression Patterns Under Salt and Drought Stress

**DOI:** 10.3390/cimb48010073

**Published:** 2026-01-11

**Authors:** Xiaojie Jin, Heping Wan, Feng Yu, Xinsun Yang, Rongchang Yang

**Affiliations:** 1 Hubei Key Laboratory of Food Crop Germplasm and Genetic Improvement, Institute of Food Crops, Hubei Academy of Agricultural Sciences, Wuhan 430064, China; xiaojiejin@hbaas.com; 2 College of Life Science, Jianghan University, Wuhan 430056, China; wanheping@jhun.edu.cn; 3 School of Life Science, Hubei University, Wuhan 430062, China; yufeng@hubu.edu.cn; 4 School of Agricultural Sciences, Murdoch University, Perth, WA 6150, Australia

**Keywords:** sweet potato, *RTE* gene family, salt stress, drought stress, gene expression profiling

## Abstract

Ethylene is a multifunctional phytohormone that regulates plant growth, development, and responses to abiotic/biotic stresses. *RTE1* (*Reversion-To-Ethylene Sensitivity1*) acts as a negative regulator of the ethylene responses in *Arabidopsis* by positively regulating ethylene receptor ETR1. However, the role of *RTE* genes in sweet potato (*Ipomoea batatas*), an import food crop worldwide, remains largely unknown, particularly their involvement in abiotic stress adaptation. In this study, we identified 23 *RTE* genes in sweet potato, distributed across 21 chromosomes and one scaffold BrgTig00017944. The phylogenetic analysis divided them into two groups, the RTE1 group and RTH (RTE1-Homolog) group. Synteny analysis revealed that whole genome duplication (WGD) was the major force of expansion of the *IbRTE* gene family. Multiple cis-acting elements responsive to hormones and stress were found in the promoter region of *IbRTE* genes. The transcriptome expression profiling showed that the majority of *IbRTEs* have tissue-specific and differential expression under drought and salt stresses. Meanwhile, the qRT–PCR results showed that the 14 representatives *IbRTEs* have differential expression profilings under salt (NaCl) and drought (PEG) treatments. These findings suggest that the *IbRTE* genes may be involved in sweet potato’s adaptive responses to salt and drought, providing a valuable foundation for further functional studies.

## 1. Introduction

Sweet potato (*Ipomoea batatas* [L.] Lam) is a dicotyledonous plant belonging to the *Convolvulaceae* family and is recognized as one of the most important root crops globally, with annual world production approaching 100 million tons [1,2]. The storage roots, as the primary harvested organ, are rich in carbohydrates, dietary fiber, vitamins, and minerals and contain a variety of health-promoting bioactive compounds such as carotenoids, anthocyanins, flavonoids, and phenolic acids [3,4]. Due to its high nutritional value, usage versatility, and resilience, sweet potato contributes significantly to food security, animal feed, industrial applications, and bioenergy production [5,6]. It is widely cultivated in over 100 countries and is mainly grown on marginal land [7]. Therefore, it is necessary to improve its salt and drought tolerance to cope with the yield losses caused by the drought and salinity of marginal land [7].

Drought is one of the most frequent and devastating abiotic stresses, characterized by unpredictability, long duration, and wide-ranging impacts on plant physiology and yield [8]. It induces multiple physiological and biochemical responses, including stomatal closure, reduced photosynthesis, accumulation of reactive oxygen species (ROS), and altered hormone signaling [8,9]. Salt stress is another major abiotic constraint that leads to drought stress, ion toxicity (especially from Na^+^ and Cl^−^), and nutrient uptake imbalances, thereby impairing plant growth and productivity [10]. To ensure stable yields under adverse conditions, a better understanding of the genetic basis of stress tolerance in sweet potato is essential. Among the major hormonal pathways involved in stress response, the ethylene signaling pathway plays a central role [11].

Ethylene is a multifunctional phytohormone that regulates numerous developmental processes, including seed germination, hypocotyl elongation, root hair formation, leaf senescence, abscission, fruit ripening, and responses to both abiotic and biotic stressors [12,13]. Ethylene signaling operates through a well-coordinated pathway in which ethylene receptor-interacting RTE proteins can modulate ethylene signaling by interacting with ethylene receptors and influencing downstream components, thereby fine-tuning plant physiological responses and environmental adaptability [13,14,15]. *RTE1* of *Arabidopsis* was isolated from a suppressor screen of the dominant ethylene-insensitive mutant *etr1-2* [16]. The AtRTE1 protein co-localizes and interacts directly with AtETR1, a key ethylene receptor, in the Golgi and endoplasmic reticulum, modulating a negative feedback loop on ethylene response adaptation [13,16,17]. The *RTE1* gene family is highly conserved in plants, animals, and protists. In *Anadiplosis*, *RTH* (*RTE1-Homolog*) is the only homolog of the *RTE1* gene family with 61.5% similarity, and acts via RTE1 in regulating ethylene response and signaling [16,18,19]. In rice, three *RTE1* homologous genes have been identified. Among them, *OsRTH1* and *OsRTH2* are clustered in the same clade as *AtRTE1*, while *OsRTH3* is grouped with *AtRTH*. However, only *OsRTH1* is involved in regulating ethylene responses in rice. Overexpression of *OsRTH1* significantly suppresses ethylene-induced developmental changes, including leaf senescence, seedling leaf elongation, coleoptile elongation and curvature, and adventitious root development [18]. In maize, six *ZmRTE* genes have been identified, and most exhibit higher expression in leaves under salt stress, suggesting a role in shoot-based stress adaptation [20]. Additionally, *ZmRTL2* and *ZmRTL4* interact with members of the ARGOS protein family in maize to modulate ethylene signaling, thereby reducing ethylene sensitivity and enhancing drought tolerance and grain yield under stress conditions [21,22]. Similarly, in cotton, *GhRTE6* has been shown to be involved in both developmental processes and salt stress responses, underscoring the diverse functionality of *RTE* genes [23].

Despite the extensive studies in other species, no comprehensive genome-wide investigation of the *RTE* gene family has been reported in sweet potato. The species is an autohexaploid (2n = B_1_B_1_B_2_B_2_B_2_B_2_ = 6X = 90) with a large and highly heterozygous genome [24]. This genomic complexity results in intricate genetic traits, which have hindered gene discovery through forward genetics approaches [24,25]. Nevertheless, it also presents unique opportunities for investigating novel gene functions and evolution processes. Therefore, a systematic analysis of *RTE* genes in sweet potato is needed to elucidate their structure, evolutionary relationships, and potential functional roles under stress conditions.

In this study, we identified 23 *IbRTE* genes in the sweet potato genome and conducted comprehensive analyses of their gene structures, conserved motifs, promoter cis-acting elements, and phylogenetic relationships. We also assessed their expression profiles under drought and salt stress using transcriptome and qRT–PCR. Our findings contribute to a better understanding of the roles of *RTE* genes in ethylene-mediated stress responses and provide a theoretical basis for breeding in stress-tolerant sweet potato.

## 2. Materials and Methods

### 2.1. Plant Materials and Stress Treatments

Sweet potato cultivar Eshu 11 was used as the experimental material for this study. Stem cuttings (~25 cm in length) with apical tips were inserted into the planting medium at a depth of ~10 cm. The cuttings were placed in Hoagland nutrient solution supplemented with either 20% PEG-6000 (*w*/*v*) or 200 mM NaCl to simulate drought and salt stress, respectively, with five plants per treatment. An additional five cuttings were placed in Hoagland solution without PEG or NaCl and used as the control group. The third unfolded leaves were collected at 7 days after planting from three random plants per treatment, representing three biological replicates. The samples were immediately flash-frozen in liquid nitrogen and stored at −80 °C until RNA extraction. All experiments were conducted in a greenhouse at the School of Life Sciences, Jianghan University, Wuhan, Hubei, China (30°30′ N, 114°9′ E). All treatment and control plants were arranged on the same shelf level, following a completely randomized block design.

### 2.2. Identification of IbRTE Genes and Analysis of Physicochemical Properties

The genome sequences of both autohexaploid sweet potato (*Ipomoea batatas* [L.] Lam.) and two diploid sweet potatoes (*Ipomoea triloba* and *Ipomoea trifida*) were downloaded from their genome database (http://sweetpotato.uga.edu/, accessed on 18 December 2025). The RTE1 and RTH (RTE1-Homolog) proteins of *Arabidopsis thaliana* were retrieved from TAIR (https://www.arabidopsis.org/, accessed on 18 December 2025) and used as queries to identify *RTE* genes in sweet potato by local BLASTP(version 2.17.0) searches. In parallel, the hidden Markov model (HMM) profile of the RTE domain (PF05608) was downloaded from the Pfam database (http://pfam.xfam.org/, accessed on 18 December 2025), and HMMER (http://www.hmmer.org/, accessed on 18 December 2025) was used to search for RTE-containing sequences in the sweet potato genome (E < 1 × 10^−10^) [26]. The results from both methods were combined, redundant sequences were removed, and candidate genes were further confirmed using the NCBI-CDD (https://www.ncbi.nlm.nih.gov/cdd/, accessed on 14 September 2025) and Pfam databases [26,27]. The physicochemical properties of the IbRTE proteins, including amino acid length, molecular weight (MW), isoelectric point (pI), instability index, aliphatic index, and grand average of hydropathicity (GRAVY), were analyzed using the ProtParam tool on ExPASy (https://web.expasy.org/protparam/, accessed on 14 September 2025) [28]. Then, subcellular localization of the IbRTE proteins were predicted using WoLF PSORT (https://wolfpsort.hgc.jp/, accessed on 18 December 2025) [29].

### 2.3. Sequence Alignment and Phylogenetic Analyses

RTE/RTH protein sequences from *Arabidopsis thaliana*, *Oryza sativa*, *Zea mays*, *Ipomoea triloba*, *Ipomoea trifida*, and *Ipomoea batatas* were aligned using Muscle, and a phylogenetic tree was constructed using the Maximum Likelihood method in IQ-TREE [30], with 1000 bootstrap replicates. The resulting phylogenetic tree was visualized using the web application ChiPlot (https://www.chiplot.online/, accessed on 18 December 2025) [31].

### 2.4. Gene Structure, Conserved Domain, and Protein Motif Analysis

The exon/intron structure information of *IbRTEs* was extracted from the genome annotation file. Conserved domains were confirmed using the NCBI-CDD and Pfam databases [26,27], and conserved motifs were identified using MEME (https://meme-suite.org/, accessed on 18 December 2025) with the motif number set to 10 [32]. Results were visualized using TBtools [33].

### 2.5. Chromosomal Localization and Synteny Analysis

The chromosomal positions of the *IbRTE* genes were retrieved from the genome annotation file, and their distribution was mapped using TBtools. The collinearity relationships within the *Ipomoea batatas* genome, as well as among the genomes of *Ipomoea batatas*, *Arabidopsis thaliana*, *Ipomoea triloba*, and *Ipomoea trifida*, were identified using MCScanX v1.0.0, and visualized by TBtools v2.311 [33,34]. The Ka (non-synonymous substitutions), Ks (synonymous substitutions), and Ka/Ks values of the *IbRTE* gene pairs were calculated using TBtools.

### 2.6. Cis-Acting Element Analysis

The 2000 bp genomic sequence upstream of the translation initiation site of each *IbRTE* was extracted from the genome data and analyzed for cis-acting elements using PlantCARE (https://bioinformatics.psb.ugent.be/webtools/plantcare/html/, accessed on 14 September 2025) [35]. Based on their functions, the cis-acting elements associated with hormone response, light response, plant growth and development, and stress response were analyzed.

### 2.7. Expression Profile Analysis

The RNA-seq data of Xushu 18 under drought, salt and normal conditions were downloaded from NCBI (accession PRJNA511028). After quality control, the clean reads were aligned to the sweet potato genome by HISAT2 [36]. Gene expression levels were calculated in FPKM using StringTie [37]. The expression profiles of *IbRTEs* in the fibrous root, stem, and leaves under the three treatments were analyzed and visualized by R package pheatmap v1.0.13.

### 2.8. qRT–PCR Validation of IbRTE Expression

Total RNA was extracted from sweet potato leaves under drought and salt stress using the FastPure Universal Plant Total RNA Isolation Kit (Vazyme, Nanjing, China). First-strand cDNA was synthesized with the HiScript^®^ II Q Select RT SuperMix with a gDNA wiper (Vazyme, Nanjing, China). Fourteen representative *IbRTEs* were selected based on their cis-acting element types and transcriptome data, and their relative expressions in leaves were validated by qRT-PCR. Specific primers for the qRT–PCR were designed using Primer Premier 5 and the *β*-*Actin* gene was used as the internal reference (Table S1). qRT–PCR was performed on an AriaMx Real-Time PCR System (Agilent Technologies Inc., Santa Clara, CA, USA) using the following conditions: 95 °C for 30 s, followed by 40 cycles of 95 °C for 5 s, and 55 °C for 1 min. The relative expression levels were calculated using the 2^–ΔΔCT^ method [38], with three biological and three technical replicates per sample.

## 3. Results

### 3.1. Identification and Characterization of the IbRTE Genes in Sweet Potato

**Figure 1:**
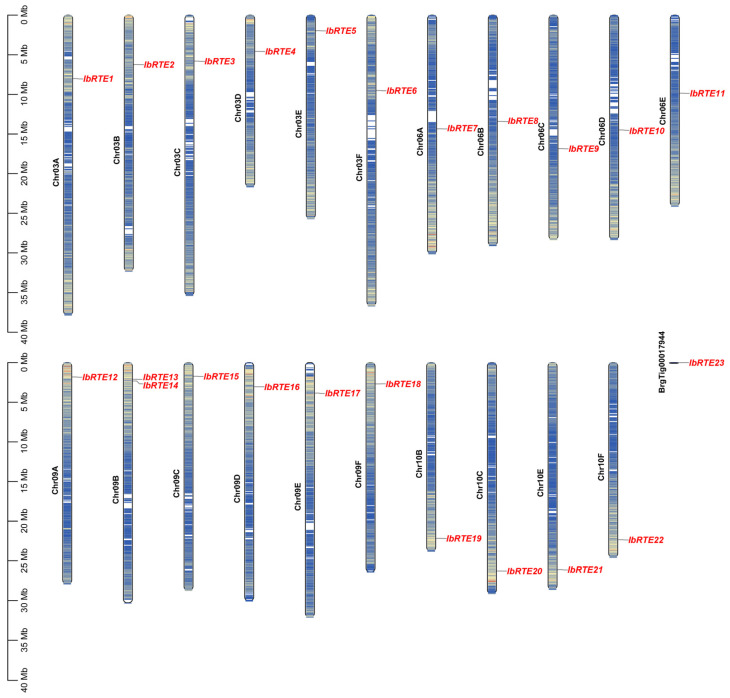
Chromosome localization of *RTE* genes in *Ipomoea batatas*.

By means of combinate BLASTP(version 2.17.0) research and HMMER research to the sweet potato genome, a total of 23 IbRTEs were identified in the sweet potato genome, and all these IbRTEs contain the conserved domains of DUF778 or the DUF778 superfamily. The genes were named *IbRTE1*–*IbRTE23* and anchored to their respective chromosomes according to their chromosomal location (Figure 1). *IbRTE23* was located on the scaffold BrgTig00017944, and its chromosomal position could not be precisely determined. The remaining genes were unevenly distributed across chromosomes 3, 6, 9, and 10. Chromosome 9 contained the largest number of genes (seven genes), followed by chromosomes 3 (six genes) and 6 (five genes), while chromosome 10 contained the fewest (four genes).

The physicochemical properties analysis revealed that the *IbRTE* genes encode proteins ranging from 98 to 386 amino acids in length, with molecular weights (MWs) between 11.08 and 44.35 kDa and theoretical isoelectric points (pI) ranging from 5.22 to 7.08. The aliphatic index varied from 82.05 to 102.32. Notably, *IbRTE1*–*IbRTE6* had an instability index > 40, indicating that these proteins may be unstable, whereas the rest had an instability index < 40, suggesting relative stability. The average hydropathicity (GRAVY) values indicated that five IbRTE proteins were hydrophilic, owing to their negative scores, whereas the remaining proteins were predicted to be hydrophobic. These results demonstrate substantial variation in the physicochemical properties of the 23 IbRTE proteins (Table 1). In addition, subcellular localization prediction results showed that most RTE-encoded proteins in sweet potato are localized to either the plasma membrane (16) or the cytoplasm (6). Notably, IbRTE20 was localized to the nucleus.

### 3.2. Phylogenetic Analysis of IbRTEs

**Figure 2:**
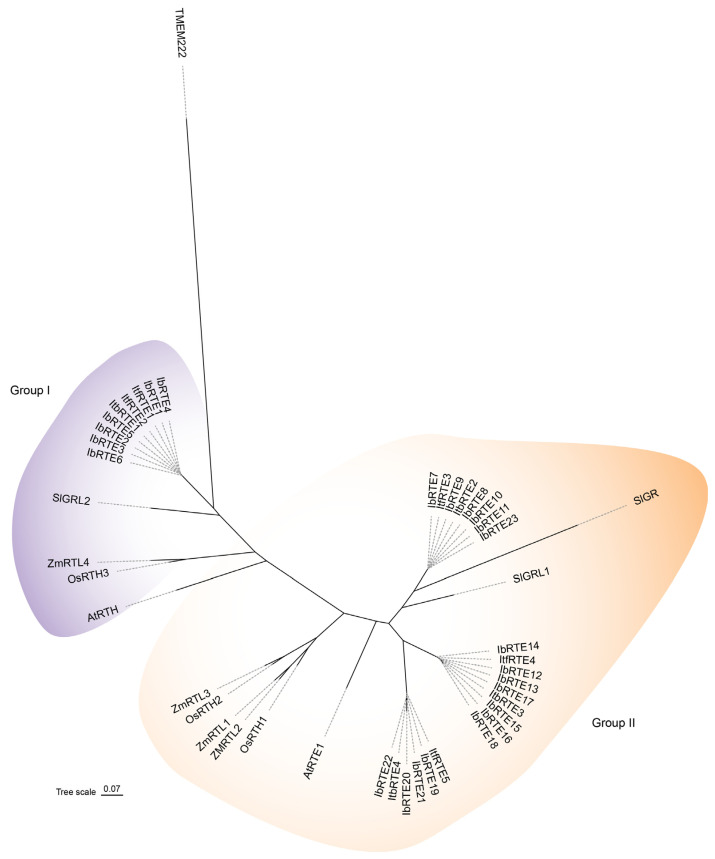
Phylogenetic tree of RTE proteins in *Ipomoea batatas* (Ib), *Arabidopsis thaliana* (At), *Oryza sativa* (Os), *Zea mays* (Zm), *Ipomoea trifida* (Itf), and *Ipomoea triloba* (Itb). TMEM222, the human homolog of RTE1, was used as an outgroup.

To explore the evolutionary relationship of IbRTEs, the homologous protein sequences from *Ipomoea batatas*, *Arabidopsis thaliana*, *Oryza sativa*, *Zea mays*, *Ipomoea trifida*, and *Ipomoea triloba* were aligned (Figure S1, Table S2), and a phylogenetic tree was constructed using the Maximum Likelihood (ML) method. The results revealed that IbRTE1-IbRTE6, OsRTH3, ZmRTL4, and AtRTH (AT3G51040) were clustered in group I, suggesting that they are RTE1-homologous genes (Figure 2). Meanwhile, IbRTE7-IbRTE23, OsRTH1, OsRTH2, ZmRTL1-ZmRTL3, three ItfRTEs, and three ItbRTEs were in group II with AtRTE1 (AT2G26070), suggesting that their roles are similar to AtRTE1. The sequence identity and similarity results showed that IbRTE1-IbRTE6 had higher identity with and similarity to AtRTH, while other IbRTEs had higher identity with and similarity to AtRTE1 (Table S3). In addition, the sequence alignments show that IbRTE proteins in the same chromosome group have the most similarity, and IbRTE1-IbRTE6 in the third chromosome group have the lowest similarity to the other IbRTE proteins (Figure S1). IbRTE23, which was located on a scaffold, has 98.97% similarity to IbRTE 11, which was located on chromosome 6E.

### 3.3. Synteny Analysis of IbRTEs

**Figure 3:**
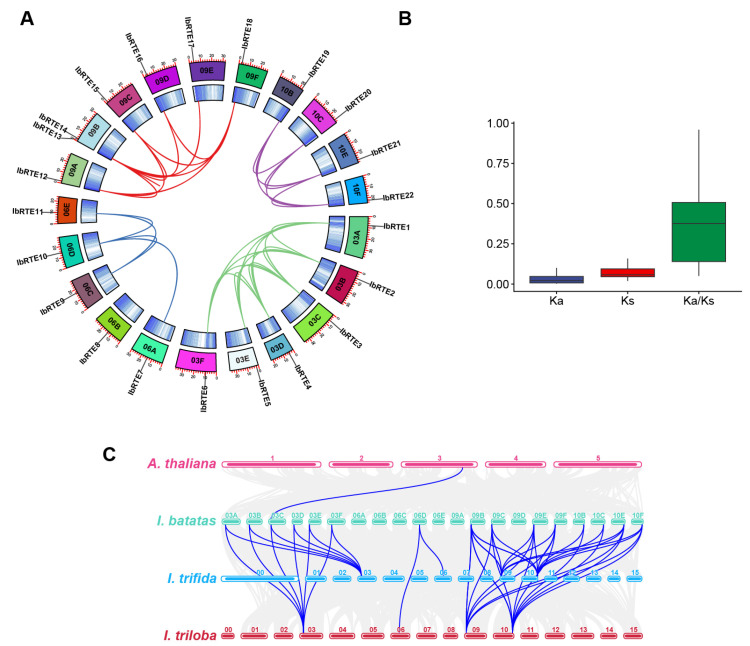
Collinearity analysis of *RTE* genes. (**A**) Intra-genomic synteny of *IbRTE* genes in sweet potato. The circles from the innermost to outermost rings represent gene density and chromosome length. (**B**) Ka, Ks, and Ka/Ks values of collinear *IbRTE* gene pairs. (**C**) Collinearity of *RTE* genes between *Ipomoea batatas* and three other species.

Intra-genomic collinearity analysis was conducted to explore the gene duplications among the 23 *IbRTE* genes. A total of 35 collinear gene pairs within the *IbRTE* family were found. Among them, only one gene pair on Chr09B, *IbRTE13*-*IbRTE14*, was identified as a tandemly duplicated gene pair. Meanwhile, 34 gene duplication events involving 21 *IbRTEs* were identified. No gene duplication in *IbRTE8* was found (Figure 3A). Interestingly, gene duplication events only occurred between *IbRTEs* in the same chromosome group, including 14 pairs in chromosome 3, 4 pairs in chromosome 6, 11 pairs in chromosome 9, and 6 pairs in chromosome 10. Some *IbRTEs* have collinearity with 2-5 other *IbRTE* genes, such as *IbRTE1* (5), *IbRTE2* (4), and *IbRTE14* (4), etc., suggesting shared structural and functional characteristics. In addition, the Ka/Ks values of the collinear gene pairs were all less than one, indicating that the *IbRTEs* were subject to purifying selection pressure (Figure 3B, Table S4).

To further explore the phylogeny and evolutionary history of *IbRTE* genes, synteny analysis between sweet potato and three other species, including *A. thaliana*, *I. trifida*, and *I. triloba*, was conducted (Figure 3C). There are 1, 16, and 16 *IbRTEs* (*IbRTE1*-*IbRTE6*, *IbRTE10*, *IbRTE13*-*IbRTE15*, and *IbRTE17*-*IbRTE22*) that show synteny with *A. thaliana*, *I. trifida,* and *I. triloba*, respectively (Figure 3C). Among them, only *IbRTE3* showed a syntenic relationship with *AtRTH* of *Arabidopsis*. A total of 23 syntenic gene pairs were found between 16 *IbRTEs* of *I. batatas* and 4 *ItfRTEs* of *I. trifida* (*ItfRTE2*, *ItfRTE3, ItfRTE4*, and *ItfRTE5*). *ItfRTE2* had a collinear relationship with *IbRTE1*-*IbRTE6*, while both *ItfRTE4* and *ItfRTE5* showed collinearity with seven *IbRTEs* (*IbRTE13*-*IbRTE15*, *IbRTE17, IbRTE18, IbRTE21*, and *-IbRTE22*) on chromosomes 9 and 10. Similarly, 23 syntenic gene pairs were found between 16 *IbRTEs* of *I. batatas* and 4 *ItbRTEs* of *I. triloba* (*ItbRTE1*, *ItbRTE2, ItbRTE3*, and *ItbRTE4*). *ItbRTE1*, *ItbRTE3*, and *ItbRTE4* displayed a collinear relationship with multiple *IbRTE* genes. These results reflect closer genome conservation and a closer evolutionary relationship among *I. batatas*, *I. trifida*, and *I. triloba*.

### 3.4. Gene Structure, Conserved Motifs, and Cis-Acting Element Analysis

**Figure 4:**
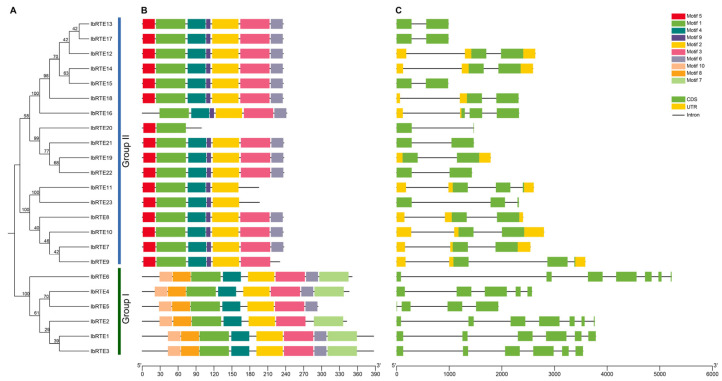
Gene structure and motif analysis of the *IbRTE* genes in sweet potato. (**A**) Phylogenetic tree of *IbRTE* genes using Neighbor-Joining method. (**B**) Conserved motifs of *IbRTE* genes. (**C**) Exon-intron structure of *IbRTE* genes.

The conserved motif analysis revealed that Motif 1 was present in all IbRTE proteins, and Motif 4, Motif 2, and Motif 3 were present in the majority (Figure 4A,B). In group I, all IbRTE proteins possessed Motif 10 and Motif 8, and most of them contained Motif 7, suggesting that these motifs may be associated with specific functions. Conversely, genes in group II lacked Motif 10, Motif 8, and Motif 7 but possessed Motif 5 and Motif 9. The exon–intron structure analysis showed that the number of exons ranges from two to seven. Genes in group II generally contained fewer exons than those in group I, including two exons (seven), three exons (seven), and four exons (three). Each gene in group I had more than four exons, and *IbRTE2* and *IbRTE6* had the largest number of exons (seven) (Figure 4A,C).

**Figure 5:**
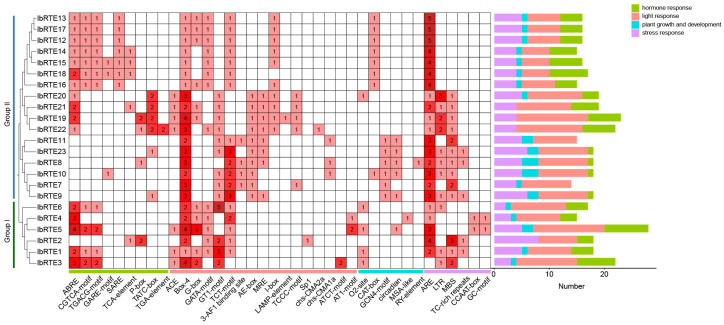
Cis-acting elements analysis of the *RTE* family genes in sweet potato. The deeper the red color, the greater the number.

To explore the potential functions and regulatory mechanisms of the *IbRTEs*, the cis-acting elements in the 2000 bp upstream promoter regions were analyzed. The prediction results revealed a rich abundance of elements related to light, stress, and hormonal responses, highlighting the potential role of the *IbRTE* genes in responding to environmental and hormonal cues (Figure 5). The stress response elements of *IbRTEs* are mainly associated with anaerobism (ARE), low-temperature (LTR), and drought (MBS), indicating that *IbRTEs* may be involved in various stress-response processes. Notably, *IbRTE12*-*IbRTE18* on chromosome 9 only contain the ARE element, suggesting their potential role in anaerobic induction responses. In addition, various types of hormone-responsive elements were predicted, including an abscisic acid (ABA)-responsive element (ABRE), jasmonic acid (JA)-responsive elements (CGTCA-motif and TGACG-motif), gibberellin (GA)-responsive elements (GARE-motif, P-box, and TATC-box), salicylic acid (SA)-responsive elements (SARE and TCA-element), and an auxin-responsive element (TGA-element). These hormones were closely associated with plant stress resistance. Therefore, the *IbRTE* genes may be widely involved in hormone-regulated stress-response pathways.

### 3.5. Expression Patterns of IbRTE Genes Under Salt and Drought Stress

**Figure 6:**
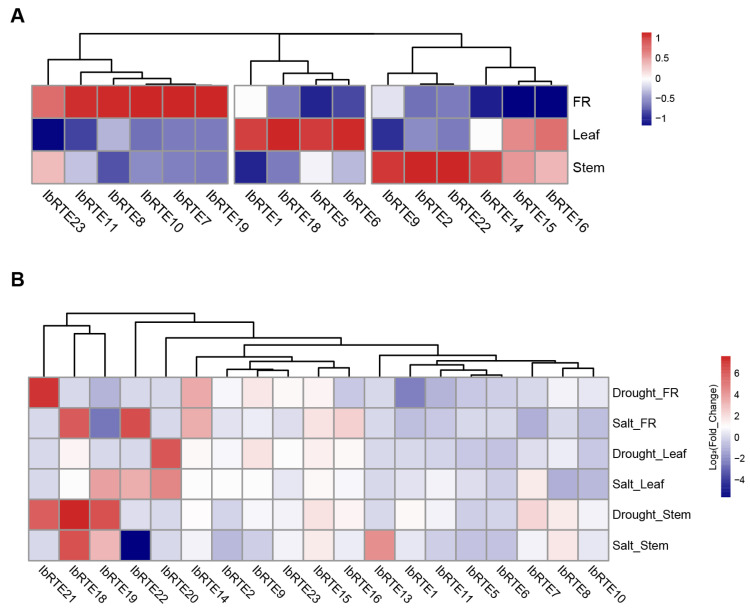
Expression pattern analysis of *IbRTE* genes under three different treatments. (**A**) The expression heatmap of 16 *IbRTEs* in three tissues under normal conditions. (**B**) The expression heatmap of 19 *IbRTEs* in three tissues under drought and salt treatment. The color scale bar represents the Log_2_(Fold_Chang) values. Red represents up-regulated expression, while blue represents down-regulated expression. FR: fibrous root, Drought_FR: comparison of fibrous root under drought and normal treatments, Drought_Leaf: comparison of leaf under drought and normal treatments, Drought_Stem: comparison of stem under drought and normal treatments, Salt_FR: comparison of fibrous root under salt and normal treatments, Salt_Leaf: comparison of leaf under salt and normal treatments, and Salt_Stem: comparison of stem under salt and normal treatments.

To investigate the functional roles of these *IbRTE* genes in response to abiotic stress, the transcriptome data of fibrous roots, leaves, and stems under normal, drought, and salt conditions were downloaded from NCBI with the accession number PRJNA511028, and analyzed. As shown in Figure 6, 16 *IbRTEs* were detected in three tissues under all three conditions. *IbRTE20* was expressed under both drought and salt stresses, while *IbRTE21* and *IbRTE13* were expressed under drought and salt stress, respectively. Under normal conditions, the expression of *IbRTEs* can be roughly classified into three patterns, including highly expressed in the fibrous root (six), leaf (four), and stem (six) (Figure 6A). Compared with normal treatment, most *IbRTEs* were up-regulated under drought and salt treatment, especially *IbRTE14*, *IbRTE15*, *IbRTE16*, and *IbRTE18* (Figure 6B). Therefore, these four *IbRTEs* were speculated to play roles in sweet potato’s responsiveness to drought and salt stresses.

**Figure 7:**
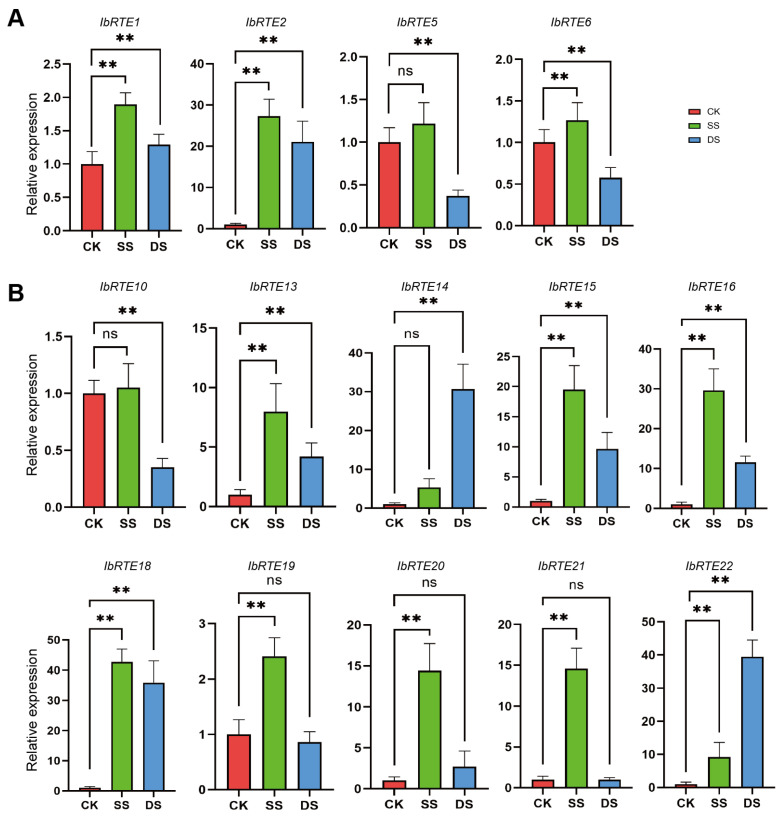
The expression patterns of 14 representative *IbRTE* genes under NaCl stress (SS) and PEG stress (DS) treatments. (**A**) The expression profiling of 4 *IbRTEs* in group I. (**B**) The expression profiling of 10 *IbRTEs* in group II. The Y-axis denotes relative expression levels calculated using the 2^−ΔΔCT^ method. CK = control, SS = NaCl (200 mM) treatment, and DS = PEG (20% *W*/*V*) treatment. Data are presented as means ± SD of three biological replicates. Statistical significance was determined using Student’s *t*-test, ** *p* < 0.01, ns: not significant.

To validate the *IbRTE* expressions, qRT-PCR was conducted to evaluate the expression profiles of 14 representative *IbRTE* genes under salt (NaCl, 200 mM) and drought (PEG, 20% *W*/*V*) treatments (Figure 7). All four *IbRTEs* (*IbRTE1*, *IbRTE2*, *IbRTE5*, and *IbRTE6*) in group I were up-regulated under salt stress, especially *IbRTE1*, *IbRTE2*, and *IbRTE6*, with significant differences. They were also significantly changed under drought stress, with two up-regulated (*IbRTE1* and *IbRTE2*) and two down-regulated (*IbRTE5* and *IbRTE6*). Among the ten *IbRTEs* in group II, the vast majority of them were significantly up-regulated under both salt and drought stresses. Notably, five *IbRTEs* in the 9th chromosome, particularly *IbRTE13*, *IbRTE15*, *IbRTE16*, and *IbRTE18*, were highly up-regulated under both abiotic stresses, suggesting their potential roles in the response to both salt and drought stresses.

## 4. Discussion

As ethylene receptor-interacting proteins, *RTE* genes negatively regulate ethylene responses and signaling in plants [13,14,15]. While the regulatory role of individual *RTE* genes has been well-documented in *Arabidopsis*, rice, maize, tomato, and other species [16,17,18,19,20,21,22,23,39,40,41], genome-wide identification and expression profiling across the *RTE* gene family remain limited in sweet potato (*Ipomoea batatas*). In this study, we identified 23 *IbRTE* genes of sweet potato (Figure 1), a number substantially higher than that reported in other species, including *Arabidopsis* (two), maize (six) and upland cotton (eight) [16,20,23]. Gene duplication represents a major force driving the expansion and evolution of gene families [42]. Sweet potato has a complex autohexaploid genome, and a whole genome duplication is considered to have occurred after crosses between progenitors [24]. The intra-genomic synteny analysis revealed that 34 paralogous pairs were found (Figure 3A, Table S4), suggesting that whole genome duplication might be the major force driving the expansion of the *IbRTE* gene family. In addition, only one tandem duplication was found, suggesting a low frequency of tandem duplication events in the expansion of the *IbRTE* gene family. Moreover, the Ka/Ks value of all *IbRTE* gene pairs were less than one (Figure 3B), indicating that they underwent purifying selection during the evolution and domestication of sweet potato. This selective pressure likely contributes to the functional conservation of the *IbRTE* gene family.

Consistent with previous phylogenetic analysis, phylogenetic analysis across *Ipomoea batatas*, *Arabidopsis thaliana*, *Oryza sativa*, *Zea mays*, *Ipomoea trifida*, and *Ipomoea triloba* grouped the IbRTEs into two major groups, the RTE1 group and RTH group [18,39,41]. Among them, IbRTE1-IbRTE6, OsRTH3, SlGRL2, and AtRTH were clustered together, whereas IbRTE7-IbRTE23, OsRTH1, OsRTH2, SlGR, and SlGRL1 were in the same group (Figure 2). Genes within the same group generally exhibited similar exon–intron structures and conserved motifs (Figure 4), indicating functional conservation. Previous studies reveal that *AtRTH* and *SlGRL2* play roles distinct from RTE1 in ethylene signaling [19,41]. Therefore, it is speculated that *IbRTE1*-*IbRTE6* may have similar functions to *AtRTH*, while *IbRTE7*-*IbRTE23* may have similar functions to *AtRTE1*.

Previous studies in *Arabidopsis* and tomato have revealed that *RTE1* and its homologs, *GR* and *GRL1*, regulate ethylene receptor activity, and thereby modulate ethylene sensitivity [40,41,43]. The overexpression of *AtRTE1*/*SlGR*/*SlGRL1* genes in these systems can enhance ethylene receptor activity, reducing sensitivity to ethylene and influencing processes such as fruit ripening and senescence [16,19,40,41]. Furthermore, the interaction of *RTE1* with ARGOS (auxin-regulated gene involved in organ size) proteins has been shown to modulate plant organ size and contribute to drought tolerance, possibly through crosstalk between the ethylene and auxin signaling pathways [21,22]. In sweet potato, promoter analysis of *IbRTE* genes revealed the presence of multiple cis-regulatory elements responsive to ABA, JA, GA, SA, and auxin, among which the most abundant elements are those related to ABA and JA (Figure 5). Both ABA and JA are crucial hormones in mediating various plant stress defense responses [44,45]. Furthermore, *IbRTEs* are enriched in stress-responsive elements, including ARE, LTR, and MBS. This suggests that *IbRTEs* may integrate into complex hormonal and environmental signaling networks and potentially participate in stress-inducible transcriptional programs. Interestingly, a disproportionately large number of light-responsive elements were distributed among the promoters. Among them, Box 4 is present in all *IbRTEs* promoters, while TCT-motif, GT1-motif, and G-box are also widely present (Figure 5). Ethylene is one of the main inducers for the breakdown of chlorophyll, which causes leaf senescence [46]. This emphasizes the possible involvement of *IbRTEs* in light-regulated developmental processes, such as photomorphogenesis or chlorophyll metabolism, which remain largely unexplored for this gene family.

To better understand the expression pattern of *IbRTEs*, their expression profiling via RNA-seq in fibrous roots, leaves, and stems under three treatments (normal, drought, and salt) were analyzed. As shown in Figure 6, 16 *IbRTE*s were tissue-specially expressed under normal conditions, while 18 and 18 *IbRTEs* were expressed under drought and salt stress, respectively. Compared to normal conditions, *IbRTE20* was expressed under both stresses, while *IbRTE21* and *IbRTE13* were expressed under drought and salt stress, respectively. Combining the results of cis-acting element analysis and transcriptome analysis, the expression profiling via qRT-PCR of 14 representative *IbRTEs* under salt and drought stresses supports the functional diversification of *IbRTE* genes in abiotic stress responses (Figure 7). The majority of them were up-regulated in response to abiotic stresses, particularly under salt stress (11 genes), mirroring trends observed in other species like maize and cotton [20,23]. Notably, *IbRTE19*, *IbRTE20*, and *IbRTE21* were significantly up-regulated under salt stress but exhibited stably expression under drought stress. Similarly, *IbRTE10* and *IbRTE14* were stable expressed under salt stress, but significantly down- and up-regulated under drought stress, respectively. This differential regulation pattern suggests that different *IbRTE* members may play distinct roles, either as primary responders or as modulators of stress response pathways. However, further functional studies are required to confirm their precise roles in response to abiotic stresses.

Taken together, these results highlight the significant role of the RTE gene family in sweet potato’s stress tolerance and adaptive growth. The combined evidence from genome-wide characterization, phylogenetics, promoter analyses, and expression studies provides a solid foundation for future functional studies, shedding light on the regulatory mechanisms by which RTE genes help plants cope with salt and drought stress.

## 5. Conclusions

In this study, a genome-wide investigation of the *RTE* family in sweet potato identified 23 *IbRTE* genes successfully mapped to 21 sweet potato chromosomes, with the exception of *IbRTE23* being unanchored. Phylogenetic and structural analyses classified these genes into two distinct groups, with highly conserved motifs and structural elements suggesting potential functional similarities within each group. Synteny analysis indicated that the vast majority of *IbRTEs* arose from whole genome duplication events. Numerous cis-regulatory elements’ responses to stress and hormones were identified in the putative promoter regions of *IbRTEs*. The expression profiling revealed that *IbRTE* genes have tissue-specific and independent expression patterns under drought and salt stresses. These findings advance our understanding of the structure, evolution, and expression patterns of the *RTE* family in sweet potato and provide valuable theoretical and genetic resources for future studies.

## Figures and Tables

**Table 1:** Physicochemical properties and subcellular localization prediction of IbRTE proteins.

Gene ID	Gene Name	Amino Acids	MW (KDa)	pI	Instability Index	Aliphatic Index	GRAVY	Subcellular Location
*IbRTE1*	*Ibat.Brg.03A_G010720.1*	386	44.17	6.43	45.59	89.43	−0.01	Plasma membrane
*IbRTE2*	*Ibat.Brg.03B_G009410.1*	341	38.44	5.22	50.96	88.09	−0.05	Plasma membrane
*IbRTE3*	*Ibat.Brg.03C_G008120.1*	386	44.35	6.94	44.55	89.43	−0.043	Plasma membrane
*IbRTE4*	*Ibat.Brg.03D_G006620.1*	345	39.35	6.7	48.47	90.99	0.063	Plasma membrane
*IbRTE5*	*Ibat.Brg.03E_G002000.1*	293	33.28	6.59	46.09	93.52	0.149	Plasma membrane
*IbRTE6*	*Ibat.Brg.03F_G012180.1*	350	40.02	5.93	45.29	94.14	0.017	Plasma membrane
*IbRTE7*	*Ibat.Brg.06A_G010150.1*	236	26.56	5.86	36.23	93.77	0.297	Cytoplasm
*IbRTE8*	*Ibat.Brg.06B_G008520.1*	235	26.46	5.86	35.9	93.74	0.267	Cytoplasm
*IbRTE9*	*Ibat.Brg.06C_G013160.1*	229	25.78	6.03	36.82	91.97	0.271	Cytoplasm
*IbRTE10*	*Ibat.Brg.06D_G009460.1*	235	26.40	5.86	36.99	94.17	0.292	Cytoplasm
*IbRTE11*	*Ibat.Brg.06E_G007670.1*	194	21.69	5.79	36.13	82.99	−0.081	Cytoplasm
*IbRTE12*	*Ibat.Brg.09A_G003520.1*	235	26.53	6.68	30.7	101.23	0.391	Plasma membrane
*IbRTE13*	*Ibat.Brg.09B_G004160.1*	235	26.53	6.68	30.7	101.23	0.391	Plasma membrane
*IbRTE14*	*Ibat.Brg.09B_G004540.1*	235	26.47	6.68	30.7	98.72	0.369	Plasma membrane
*IbRTE15*	*Ibat.Brg.09C_G003400.1*	235	26.50	6.68	31.06	98.72	0.359	Plasma membrane
*IbRTE16*	*Ibat.Brg.09D_G004680.1*	241	27.33	6.68	33.26	102.32	0.422	Plasma membrane
*IbRTE17*	*Ibat.Brg.09E_G004640.1*	235	26.53	6.68	30.7	101.23	0.391	Plasma membrane
*IbRTE18*	*Ibat.Brg.09F_G004860.1*	235	26.49	6.73	31.44	101.23	0.386	Plasma membrane
*IbRTE19*	*Ibat.Brg.10B_G022940.1*	236	26.79	7.08	24.2	87.54	0.328	Plasma membrane
*IbRTE20*	*Ibat.Brg.10C_G024310.1*	98	11.08	6.69	27.61	96.43	0.169	Nucleus
*IbRTE21*	*Ibat.Brg.10E_G021960.1*	236	26.88	6.8	24.15	87.54	0.333	Plasma membrane
*IbRTE22*	*Ibat.Brg.10F_G021320.1*	236	26.81	7.08	23.84	89.19	0.35	Plasma membrane
*IbRTE23*	*Ibat.Brg.S051350.1*	195	21.81	5.79	34.43	82.05	−0.082	Cytoplasm

## Data Availability

The original contributions presented in this study are included in the article/Supplementary Materials. Further inquiries can be directed to the corresponding authors.

## Supplementary Materials

The following supporting information can be downloaded at: https://www.mdpi.com/article/10.3390/cimb48010073/s1.
