# Supplementary material for: Genome-Wide Identification of RTE Gene Family Members in Sweet Potato and Their Expression Patterns Under Salt and Drought Stress
Source: Curr Issues Mol Biol. 2026 Jan 11;48(1):73. doi: 10.3390/cimb48010073 (PMC12839647; doi:10.3390/cimb48010073)
Supplement: Supplementary file 1 [file cimb-48-00073-s001.zip › Supplementary tables.pdf]

Table S1. Primers for qRT-PCR in RTE gene family of sweet potato.

Primer name	Forward sequence (5′-3′)	Reverse sequence (5′-3′)
q-*IbRTE1*	GCCATCCACTCCAGTATCCG	GTTCCAATCCATTGATCCACC
q-*IbRTE2*	AGTGCTGCTTTCCCCCAC	GGCGTCGTCCCAAGTGAT
q-*IbRTE5*	TGGGCATTCGTGCAACCA	TGCGAACGAGTGGCTGTT
q-*IbRTE6*	CAAACCTCGCTGGGCATT	TGGCGGATACTGGAGTGGA
q-*IbRTE10*	TAGCACGCGCCACTTTGA	CAGCCCATTGATCCGCCA
q-*IbRTE13*	TCGCCACTTTGAGCACCA	CACCCCATTGATCCGCCA
q-*IbRTE14*	GTCCTTTCCCCTGGGCAC	GCAACACGGGAACTTCGC
q-*IbRTE15*	GATGCCATCCACTCCAGTATCC	CCAGTGCCCCTTGAGCAATAT
q-*IbRTE16*	TTCCTCCAAACCTCGCCG	AGTGGCTGTTGCACGTGA
q-*IbRTE18*	AACCGGTTGGGCTTCCAG	CGCCAAAGGTGAGCCCTA
q-*IbRTE19*	GCACAGGCTGCGTTTCCA	ATGCAGCAGGGAAAGCGT
q-*IbRTE20*	AACAGGCTGCGCTTCCAT	CGATGCAGCAGGGAAAACG
q-*IbRTE21*	TGCGTGTGACGAGGACAG	ATGGAAACGCAGCCTGTG
q-*IbRTE22*	ATCGTGTGGACACCGCTG	GCTGGTGCTCCAAACGCA
β-Actin	AGCAGCATGAAGATTAAGGTTGTAGCAC	TGGAAAATTAGAAGCACTTCCTGTGAAC

Table S2. Accession numbers of *RTE* genes in *Arabidopsis*, rice, maize, *I. triloba*, and *I. trifida*.

Gene Name	Gene ID	Species
AtRTE1	AT2G26070	*Arabidopsis thaliana*
AtRTH1	AT3G51040	*Arabidopsis thaliana*
OsRTH1	Os01g0711600	*Oryza sativa*
OsRTH2	Os05g0539800	*Oryza sativa*
OsRTH3	Os03g0799500	*Oryza sativa*
ZmRTL1	GRMZM5G832994	*Zea mays*
ZmRTL2	GRMZM2G077293	*Zea mays*
ZmRTL3	GRMZM2G121208	*Zea mays*
ZmRTL4	GRMZM2G039592	*Zea mays*
ItbRTE1	itb03g07290.t1	*Ipomoea triloba*
ItbRTE2	itb06g08170.t1	*Ipomoea triloba*
ItbRTE3	itb09g04180.t1	*Ipomoea triloba*
ItbRTE4	itb10g22360.t1	*Ipomoea triloba*
ItfRTE1	itf00g45640.t1	*Ipomoea trifida*
ItfRTE2	itf03g07230.t1	*Ipomoea trifida*
ItfRTE3	itf06g10170.t1	*Ipomoea trifida*
ItfRTE4	itf09g03820.t1	*Ipomoea trifida*
ItfRTE5	itf10g22240.t1	*Ipomoea trifida*

Table S3. Protein sequence identity and similarity of RTEs and RTHs compared with AtRTE1 and AtRTH.

Protein ID	Identity (%)		Similarity (%)	
	AtRTE1	AtRTH	AtRTE1	AtRTH
OsRTH2	54.24	45.15	68.22	59.49
ZmRTL3	53.39	43.64	68.22	59.32
OsRTH1	52.19	43.7	66.14	57.56
ZmRTL1	52.99	45.99	66.93	58.23
ZMRTL2	55.08	46.19	69.07	58.9
IbRTE11	57.87	40.91	75.63	58.08
IbRTE23	57.58	41.21	75.25	58.29
IbRTE9	58.62	40.77	75.43	58.37
IbRTE8	58.47	40.76	76.27	59.24
IbRTE7	58.65	41.42	75.95	59.41
IbRTE10	58.90	41.18	75.85	58.82
IbRTE20	61.00	47.47	67.00	61.62
IbRTE21	63.14	44.35	71.61	58.16
IbRTE19	62.71	43.51	71.19	57.74
IbRTE22	63.14	43.51	71.61	57.74
IbRTE16	62.50	45.23	75.00	58.51
IbRTE18	62.34	47.66	74.48	60.00
IbRTE12	62.34	47.23	74.9	60.00
IbRTE13	62.34	47.23	74.9	60.00
IbRTE17	62.34	47.23	74.9	60.00
IbRTE14	63.60	47.66	75.31	60.43
IbRTE15	63.18	46.81	75.31	60.43
IbRTE2	38.49	51.29	52.38	65.09
IbRTE6	39.29	51.72	53.17	65.52
IbRTE1	38.89	52.16	52.78	65.95
IbRTE3	38.89	52.16	52.78	65.95
IbRTE4	40.48	54.31	54.76	68.53
IbRTE5	40.48	54.31	54.76	68.53
AtRTH	44.77	/	58.16	/
OsRTH3	47.47	55.20	62.67	63.80
ZmRTL4	47.93	53.39	63.59	64.25

At: *Arabidopsis thaliana*, Os: *Oryza sativa*, Ib: *Ipomoea batatas*, Zm: *Zea mays*.

Table S4. The Ka, Ks values of *IbRTE* syntenic gene pairs in sweet potato.

Syntenic gene pairs		Ka	Ks	Ka/Ks
IbRTE1	*IbRTE2*	0.098	0.131	0.747
IbRTE1	*IbRTE3*	0.005	0.019	0.237
IbRTE1	*IbRTE4*	0.047	0.117	0.403
IbRTE1	*IbRTE5*	0.063	0.102	0.617
IbRTE1	*IbRTE6*	0.047	0.083	0.567
IbRTE2	*IbRTE3*	0.100	0.158	0.632
IbRTE2	*IbRTE4*	0.061	0.111	0.544
IbRTE2	*IbRTE5*	0.046	0.048	0.959
IbRTE2	*IbRTE6*	0.020	0.040	0.505
IbRTE3	*IbRTE4*	0.052	0.120	0.435
IbRTE3	*IbRTE5*	0.061	0.113	0.538
IbRTE3	*IbRTE6*	0.046	0.097	0.469
IbRTE4	*IbRTE5*	0.010	0.059	0.169
IbRTE4	*IbRTE6*	0.055	0.116	0.474
IbRTE7	*IbRTE10*	0.002	0.032	0.056
IbRTE9	*IbRTE10*	0.011	0.027	0.427
IbRTE9	*IbRTE11*	0.029	0.077	0.373
IbRTE10	*IbRTE11*	0.031	0.085	0.362
IbRTE12	*IbRTE14*	0.006	0.049	0.114
IbRTE12	*IbRTE15*	0.006	0.043	0.130
IbRTE12	*IbRTE18*	0.007	0.059	0.110
IbRTE13	*IbRTE14*	0.006	0.049	0.114
IbRTE13	*IbRTE17*	0.000	0.000	\
IbRTE14	*IbRTE15*	0.004	0.056	0.067
IbRTE14	*IbRTE16*	0.023	0.069	0.33
IbRTE14	*IbRTE18*	0.008	0.046	0.182
IbRTE14	*IbRTE17*	0.006	0.049	0.114
IbRTE15	*IbRTE18*	0.008	0.072	0.117
IbRTE16	*IbRTE18*	0.020	0.052	0.377
IbRTE19	*IbRTE20*	0.023	0.045	0.507
IbRTE19	*IbRTE21*	0.016	0.041	0.390
IbRTE19	*IbRTE22*	0.002	0.037	0.050
IbRTE20	*IbRTE21*	0.043	0.053	0.814
IbRTE20	*IbRTE22*	0.023	0.060	0.376
IbRTE21	*IbRTE22*	0.014	0.041	0.343
