# Supplementary figures and images for: Genome-Wide Identification of RTE Gene Family Members in Sweet Potato and Their Expression Patterns Under Salt and Drought Stress
Source: Curr Issues Mol Biol. 2026 Jan 11;48(1):73. doi: 10.3390/cimb48010073 (PMC12839647; doi:10.3390/cimb48010073)

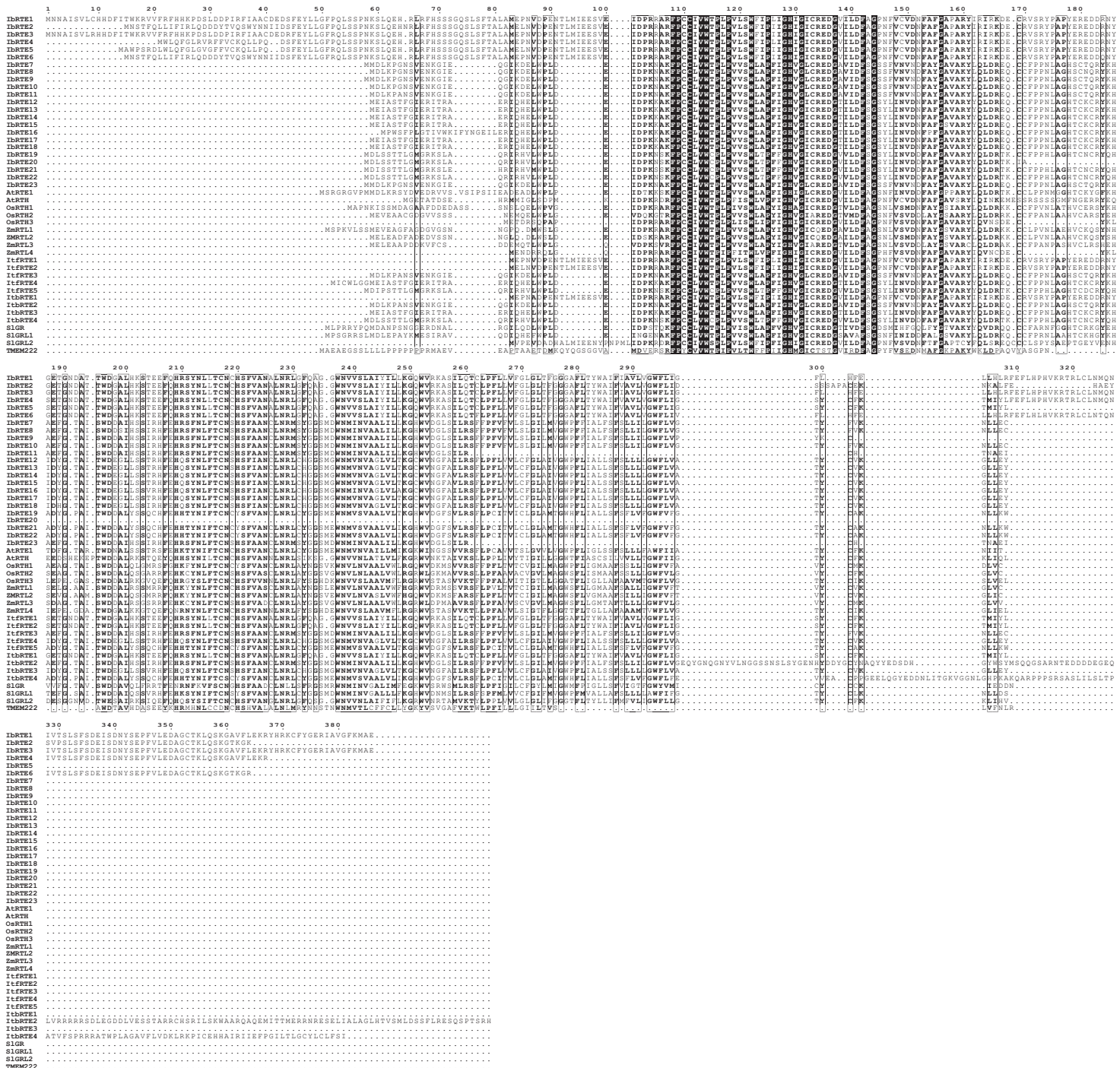

Supplement: Supplementary file 1 [file cimb-48-00073-s001.zip › Figure S1.pdf]
